# A Broadband Terahertz Waveguide T-Junction Variable Power Splitter

**DOI:** 10.1038/srep28925

**Published:** 2016-06-29

**Authors:** Kimberly S. Reichel, Rajind Mendis, Daniel M. Mittleman

**Affiliations:** 1 School of Engineering, Brown University, Providence, Rhode Island 02912, USA

## Abstract

In order for the promise of terahertz (THz) wireless communications to become a reality, many new devices need to be developed, such as those for routing THz waves. We demonstrate a power splitting router based on a parallel-plate waveguide (PPWG) T-junction excited by the TE_1_ waveguide mode. By integrating a small triangular septum into the waveguide plate, we are able to direct the THz light down either one of the two output channels with precise control over the ratio between waveguide outputs. We find good agreement between experiment and simulation in both amplitude and phase. We show that the ratio between waveguide outputs varies exponentially with septum translation offset and that nearly 100% transmission can be achieved. The splitter operates over almost the entire range in which the waveguide is single mode, providing a sensitive and broadband method for THz power splitting.

As the demand for wireless bandwidth increases, the use of terahertz (THz) frequencies for wireless communications has experienced a surge of attention. Numerous recent reviews discuss a growing list of options for THz transceivers, evaluation of THz wave propagation windows through the atmosphere, and possible network architectures^1^,^2^,^3^. However, many important system needs remain unaddressed. For example, there has been little attention directed towards the need for routing and switching of THz light^3^. One of the most basic routers is a switch that can pass a signal from one input channel to multiple output channels^4^. A useful example of such a switch is a guided wave T-junction power splitter, a geometry that is well known in both the microwave^5^,^6^,^7^ and optical^8^,^9^ regimes. In the THz range, similar configurations have been proposed, including slot waveguides^10^, planar Goubau lines^11^, and photonic crystals^12^. Due to the inherent limitations of these geometries, however, the ratio of power splitting is typically fixed^10^. In THz photonic crystal power splitters, an external magnetic field can be used to vary the coupling ratio^12^,^13^,^14^, a novel idea which however adds considerable complexity and cost.

Here, we demonstrate a variable power splitter based on a movable septum integrated into a parallel-plate waveguide (PPWG) H-plane T-junction. We use the TE_1_ waveguide mode which has already been employed for a promising number of THz applications^15^,^16^,^17^,^18^,^19^. The device splits the propagating THz wave from one input waveguide channel to two output waveguide channels. By simple mechanical translation of the septum, we can vary the ratio of power at the two output ports, without causing any additional misalignment to the waveguide configuration. The device operates with a continuously variable power ratio over nearly the entire single-mode region of the waveguide. Indeed, it can even be used over a much larger bandwidth as long as frequencies near the higher-order mode cutoff frequencies are avoided. Thus, the device provides a versatile and robust broadband solution to one of the key challenges in THz signal routing.

## Device Design

**Figure 1:**
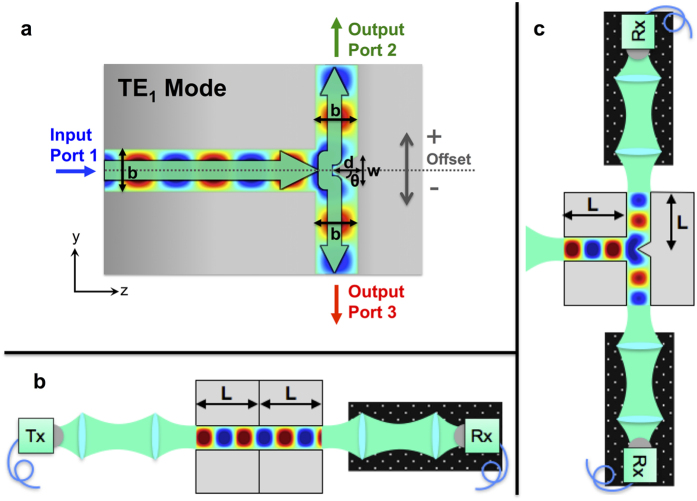
T-Junction Design and Experimental Setup. (**a**) Diagram of PPWG H-plane T-junction with tunable septum. Each arm has a plate separation of *b* = 1 mm. The septum is a triangular shape with angle of *θ* = 30° as measured from the tip away from the center. For a septum depth, *d* = 600 μm, this gives a width, *w* = 692.8 μm. The offset is varied in steps of 100 μm from −700 μm to +700 μm by translating the whole metal piece, where we define positive offsets to be when the septum is closer to Port 2. Experimental setup where (**b**) shows the reference configuration and (**c**) shows the T-junction configuration. The length, *L*, depicted here is 12.7 mm and the input excitation in (**c**) is the same as shown in (**b**).

**Figure 2:**
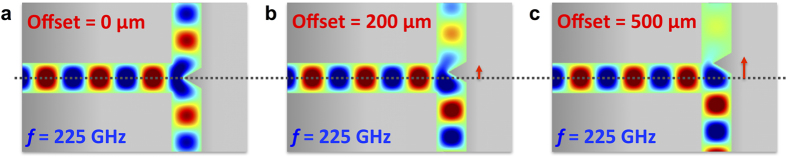
FEM Simulation Demonstrating T-Junction Operation. Simulation showing operating principal of variable T-junction device excited by the TE_1_ waveguide mode at a selected frequency of 225 GHz. The red arrows illustrate the offset distance of (**a**) 0 μm, (**b**) 200 μm, and (**c**) 500 μm. As the septum is moved less transmission can be seen through Port 2 and stronger coupling can be seen through Port 3. The change in phase with septum offset can also be clearly seen.

The T-junction design is depicted in Fig. 1(a). We excite the PPWG with the TE_1_ waveguide mode at the bottom of the “T”, so this is an H-plane T-junction (i.e., the guided wave’s magnetic field polarization axis is in the plane of the “T”)^20^. The T-junction serves as a power splitter, taking light from one input channel and splitting it into the two perpendicular output channels. Typically the junction consists of a flat wall and the power is split equally between either output arm^7^,^10^. Here, we incorporate a triangular septum that juts out from this junction wall. Septa have been used in microwave T-junctions^21^,^22^, but because the rectangular waveguide geometry has walls on each side, the septum position is typically fixed, and therefore cannot be used as a tuning element. In our case, we are free to translate the septum by moving the metal plate with the septum relative to the other two (fixed) waveguide walls, since the PPWG is open on two sides. Figure 2 demonstrates the operating principle of the translatable septum. The septum acts as a rudder to direct light down either one pathway or the other, thereby giving the ability to tune the power splitting ratio between the two output arms. The triangular shape of the septum, as well as its height and base width, are optimized using numerical simulations in order to minimize back-reflection towards the input port and to maximize the sensitivity of the splitting ratio to the septum’s position relative to the input waveguide’s symmetry plane.

We anticipate that the most common implementation of this splitter will be for operation in the single-mode range of the waveguide. For a plate separation of *b* = 1 mm, the waveguide is restricted to the single TE_1_ mode in the range from 150–300 GHz, thus providing a full octave of operating bandwidth.

The experimental device is fabricated from bulk aluminum using standard machine milling. Figure 1(a) shows a 2D slice of our device in the y-z plane. The device extends 5 cm in the x-direction (out of the plane of the diagram) so that the plates are wide enough in the unconfined direction to ensure that no edge effects are present. We characterize our device (Fig. 1(b–c)) using a commercial pulsed THz system using fiber coupled photoconductive antennas for THz generation and detection. We use two lenses after the transmitter to focus down onto our waveguide with a 1/*e* diameter of 4 mm at 300 GHz. We use the same collection optics at the output of the waveguide.

We measure the signal at both output ports as a function of the position of the septum, in 100 μm increments, and use a Savitzky-Golay smoothing filter on the experimental data to remove some of the system noise. In order to obtain a reference waveform for normalization of these measured signals, we use a straight waveguide with a path length equal to that of the input arm plus one output arm (Fig. 1(b)). This accounts for the fact that the beam diffracts slightly in the unconfined (out of the plane of the diagram) direction, an effect that accumulates linearly with increasing propagation distance inside the waveguide. There are also ohmic losses that grow with increased propagation, but this value is small. This procedure ensures an accurate comparison of the input spectrum to the spectrum emerging from each of the output arms. The output collection optics and receiver are on a breadboard so that it can be easily moved between output channels while still maintaining the same light collection configuration (Fig. 1(c)). The center position of the septum is determined by comparison of the THz signal emerging from the two output ports – when they are equal, we assume that the septum is accurately centered. The plate separation of the output arms is verified by comparing the output of each arm when the septum is translated to the position of maximum transmission – at the correct plate separation the waveguide cutoff frequency matches that of the reference.

### Variable Power Splitting Performance

**Figure 3:**
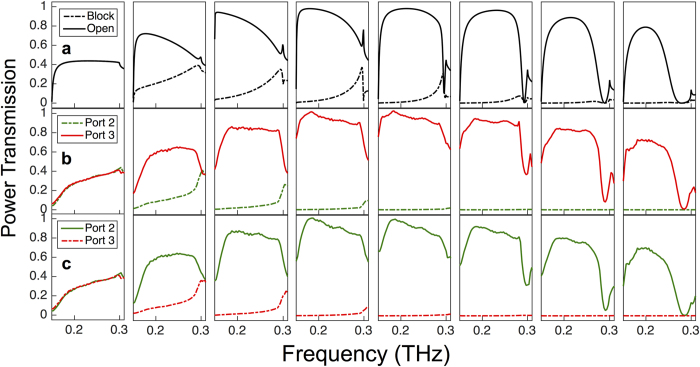
T-Junction Performance. Power transmission as a function of frequency for septum offsets increasing from left to right in steps of 100 μm, where the solid lines represent the output channel that is open and the dashed lines represent the output channel that is blocked. The top panels (**a**) show simulation results, (**b**) experimental results for offsets from 0 μm to +700 μm where the septum directs the light to the Port 3 output (shown in red), and (**c**) experimental results for offsets from 0 μm to −700 μm where the septum directs the light to the Port 2 output (shown in green).

We compare the experimental measurements to finite-element method (FEM) simulation for septum offsets of −700 μm to +700 μm (where we define the positive offset to be in the direction as shown in Fig. 1(a)) in steps of 100 μm, shown in Fig. 3. Since simulations are perfectly symmetric, Fig. 3(a) shows simulation results where the solid lines indicate the port that the light is directed towards, and the dashed lines indicate the port that is more blocked. Figure 3(b) shows experimental results for positive offsets where light is directed down Port 3, and Fig. 3(c) shows results for negative offsets where light is directed down Port 2. When the offset is zero (left panels of Fig. 3), the power is split equally between Port 2 and Port 3 (about 40% in each output arm, with the remaining 20% back-reflected from the septum tip). As we translate the septum, the back-reflection decreases and we find the ratio between output arms (Port 3 : Port 2, or vice versa) to be extremely sensitive to the offsets close to the zero position. At a central frequency of 225 GHz, we observe the coupling ratio vary from 1:1 for offset = 0 μm, to 6:1 for offset = ±100 μm, and to 30:1 for offset = ±200 μm. The maximum output occurs at offset = ±400 μm where we achieve almost 100% coupling (and therefore nearly zero back-reflection), then begins to decrease for larger offsets. These trends are clearly visible in both experiment and simulation.

**Figure 4:**
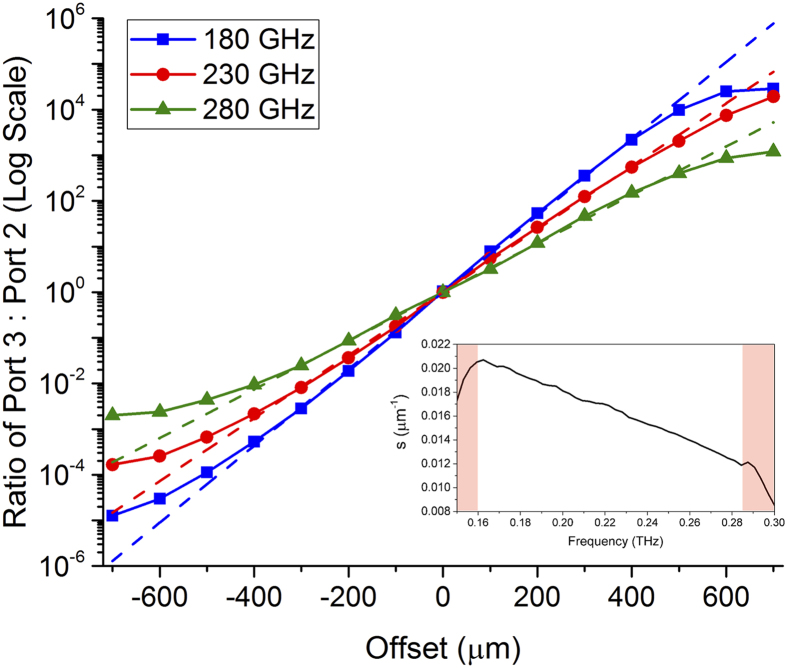
Power Splitting Ratio. Ratio between waveguide outputs for Port 3: Port 2 from experimental measurements on a log scale at a few selected frequencies of 180 GHz (blue squares), 230 GHz (red circles), and 280 GHz (green triangles). A linear fit to the region from −400 μm to +400 μm shows the relationship *ratio* = *exp*{± *s* · *offset*} plotted as the dashed lines. The inset shows *s* as a function of frequency, which is linear except for the regions near cutoffs shaded in red.

To quantify our results, we take the ratio of the power between output channels for a few selected frequencies as a function of the septum offset, shown in Fig. 4, plotted on a log scale. From the range of −400 μm to +400 μm (from maximum transmission through Port 2 to maximum transmission in Port 3), the ratio between waveguide output arms varies exponentially. By fitting to this central portion of the data, we extract a power splitting coefficient, *s*, defined by *ratio* = *exp*{± *s* · *offset*}. We plot *s* as a function of frequency in the inset of Fig. 4. The parameter *s* shows the rate at which frequencies prefer one output channel over the other. This analysis shows that lower frequencies are more sensitive to the offset position and are diverted more quickly.

**Figure 5:**
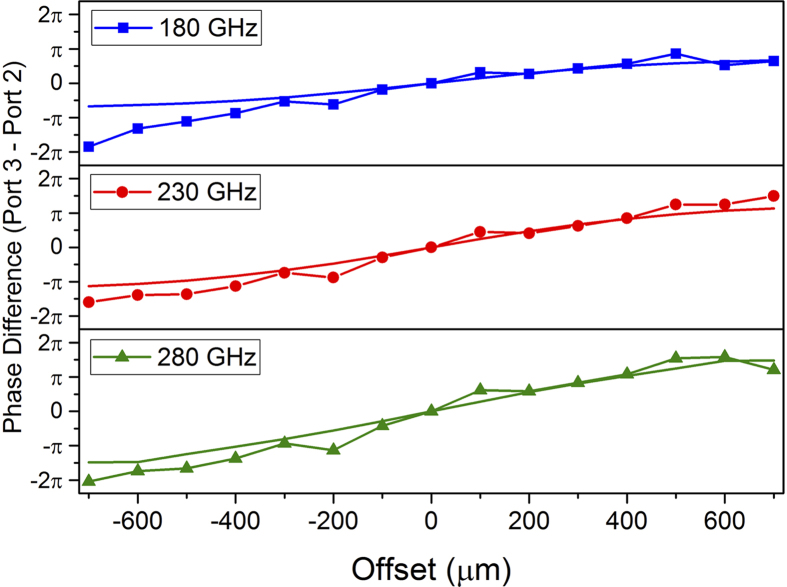
Phase Difference as a Function of Septum Offset. Phase difference between Port 3 and Port 2 from experiment as a function of septum offset for three selected frequencies of 180 GHz (blue squares), 230 GHz (red circles), and 280 GHz (green triangles). The solid lines are from simulation. The slope of the phase difference increases with increasing frequency.

In addition to the power ratio, we extract the phase at the output ports, which is an important parameter in many applications, such as for phased array antennas^23^. When the septum is centered, the phase at Port 2 and Port 3 is equal. However, when the septum is offset, the phase difference increases simply because the wave propagates a different distance down each output arm. The experimental and simulated phase difference between Port 3 and Port 2 as a function of septum offset is shown in Fig. 5 for a few selected frequencies. The slope of the phase difference increases slightly with increasing frequency, since the group velocity increases with increasing frequency for the TE_1_ waveguide mode^16^. The shift in phase can be visualized by comparing the field pattern at the output of Port 3 in Fig. 2(a) and Fig. 2(c). The phase at each output could be matched by changing the length or width of the output arm, as has been demonstrated in rectangular waveguide T-junctions^22^. However, since rectangular waveguides geometries are fixed, the power splitting ratio would not be variable in these configurations. Using the PPWG that is open on two sides, one could also incorporate a variable width of the output waveguide plate separation in order to provide a method for fine-tuning of the phase.

## Discussion

This first design of a tunable septum depends on translating the entire metal section with the septum. Here, this has been accomplished manually; however, a motorized automated tuning could be easily implemented. Additional tuning capability can be incorporated by adjustment of the plate separation, either at the input port or (independently) at the two output ports. As the plate separation decreases the cutoff frequency increases, shifting the device operation to higher frequencies, at the expense of possibly reducing the input and output coupling between the guided wave and free space. We also note that our splitter does function at higher frequencies above the single-mode region with dips approaching each cutoff of the higher-order modes. At these higher frequencies, the power is distributed across all the excited modes, and therefore all modes must be considered to account for the output transmission.

In conclusion, we have demonstrated a H-plane T-junction with translatable septum that functions as a variable power splitter. The power splitting ratio between output arms varies exponentially and nearly 100% transmission can be achieved. This coupling is highly controllable in both amplitude and phase, and the device can be used over practically the entire single mode region. This demonstrates a key component for signal routing of THz waves, which will be important for future applications in wireless networking.

## Additional Information

**How to cite this article**: Reichel, K. S. *et al*. A Broadband Terahertz Waveguide T-Junction Variable Power Splitter. *Sci. Rep.* **6**, 28925; doi: 10.1038/srep28925 (2016).
